# Supplementary material for: A Combination of Alectinib and DNA-Demethylating Agents Synergistically Inhibits Anaplastic-Lymphoma-Kinase-Positive Anaplastic Large-Cell Lymphoma Cell Proliferation
Source: Cancers (Basel). 2023 Oct 21;15(20):5089. doi: 10.3390/cancers15205089 (PMC10605931; doi:10.3390/cancers15205089)
Supplement: Supplementary file 1 [file cancers-15-05089-s001.zip › cancers-2402547-File S1.pdf]

Figure 2 b_Karpas299

Membrane(stained with ponceau-S)

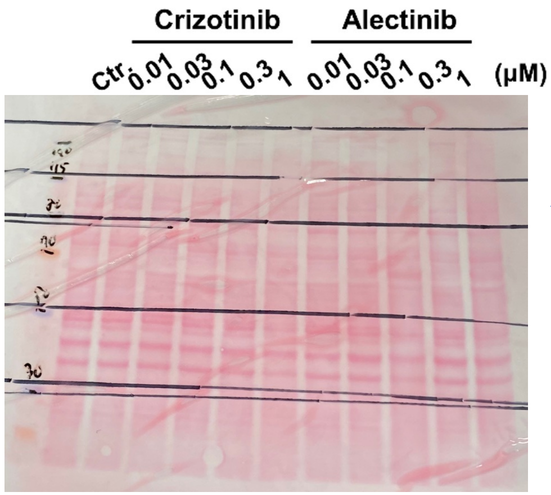

Original

pSTAT3

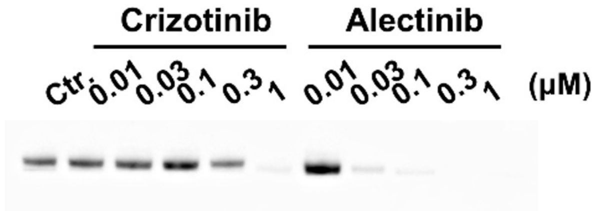

※Signal intensity 1 1.17 1.24 1.60 0.78 0.18 2.35 0.20 0.17 0.13 0.12

Marged with marker

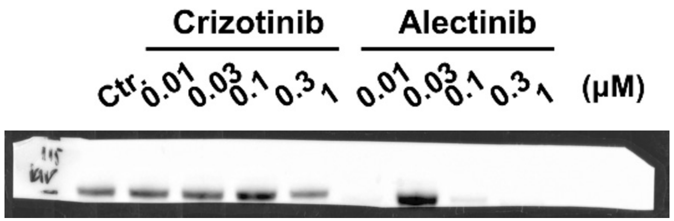

Figure 2 b_Karpas299

Membrane(stained with ponceau-S)   **Original**   **Marged with marker**

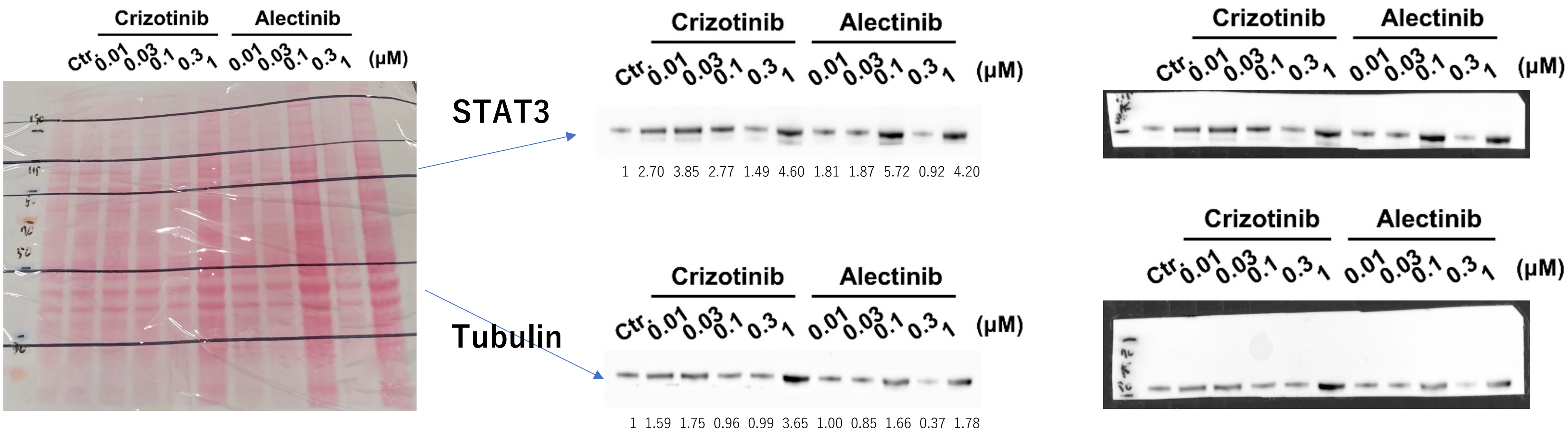

Figure 2 b_SR786

Membrane(stained with ponceau-S)

Original

Marged with marker

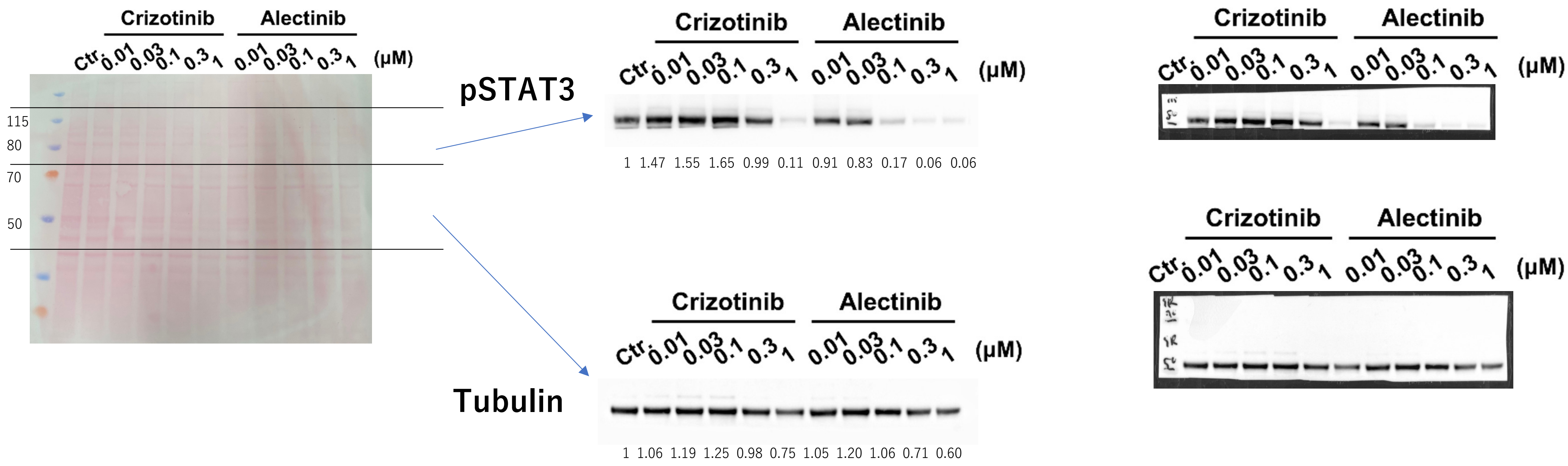

Figure 2 b_SR786

Membrane(stained with ponceau-S)

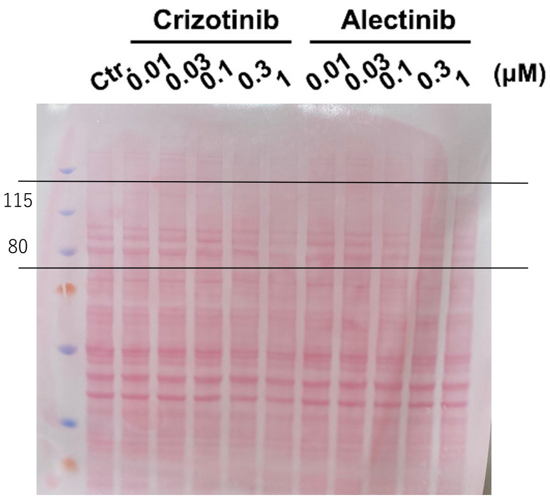

Original

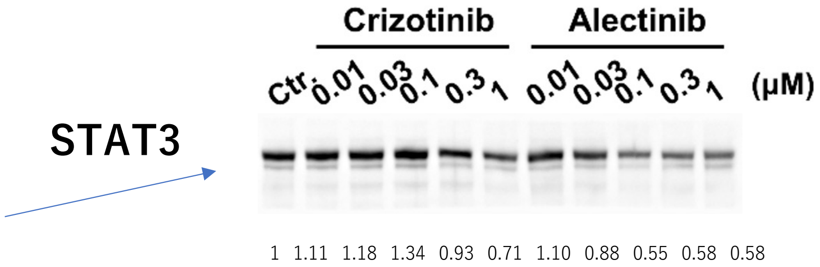

Marged with marker

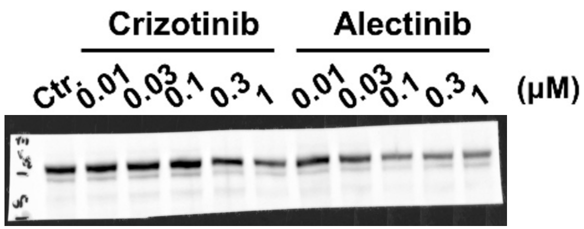

Figure 4 b

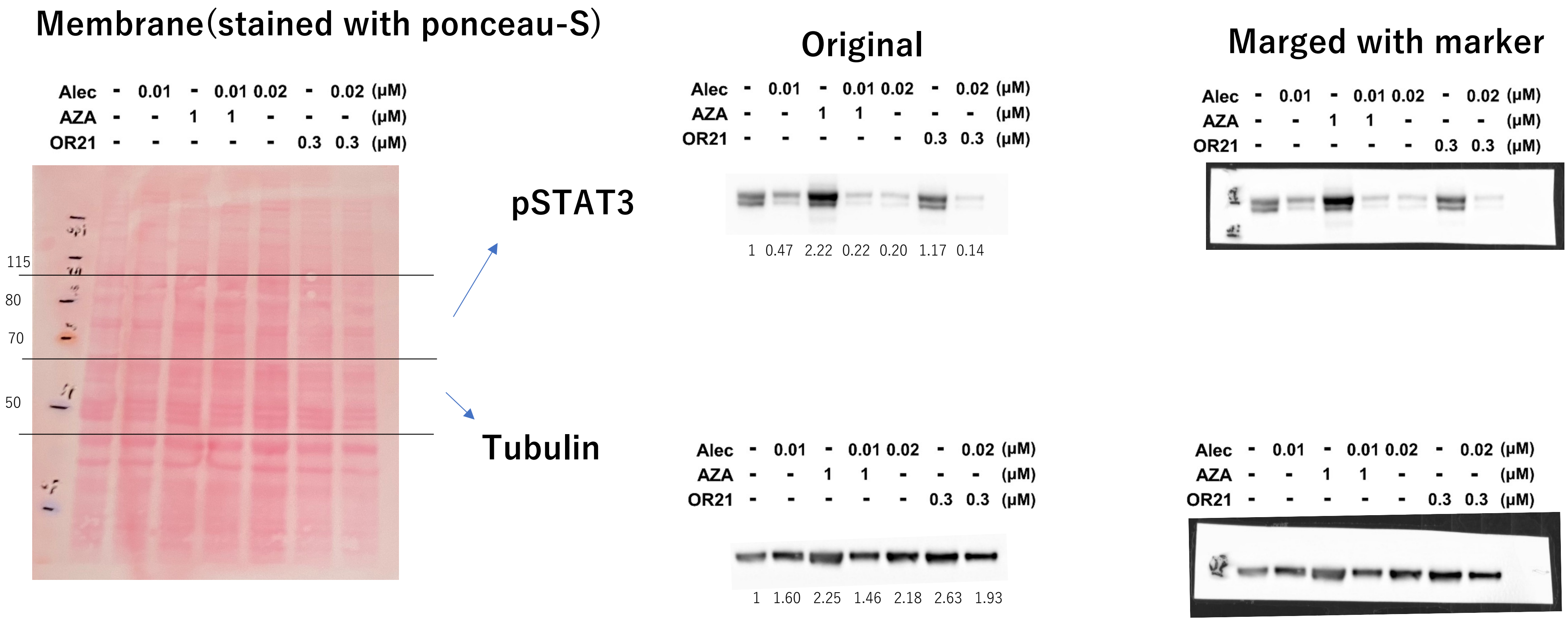
Membrane(stained with ponceau-S)
Alec - 0.01 - 0.01 0.02 - 0.02 (μM)
AZA - - 1 1 - - - (μM)
OR21 - - - - - 0.3 0.3 (μM)
115
80
70
50
pSTAT3
Tubulin
Original
Alec - 0.01 - 0.01 0.02 - 0.02 (μM)
AZA - - 1 1 - - - (μM)
OR21 - - - - - 0.3 0.3 (μM)
1 0.47 2.22 0.22 0.20 1.17 0.14
Alec - 0.01 - 0.01 0.02 - 0.02 (μM)
AZA - - 1 1 - - - (μM)
OR21 - - - - - 0.3 0.3 (μM)
1 1.60 2.25 1.46 2.18 2.63 1.93
Marged with marker
Alec - 0.01 - 0.01 0.02 - 0.02 (μM)
AZA - - 1 1 - - - (μM)
OR21 - - - - - 0.3 0.3 (μM)
Alec - 0.01 - 0.01 0.02 - 0.02 (μM)
AZA - - 1 1 - - - (μM)
OR21 - - - - - 0.3 0.3 (μM)

Figure 4 b

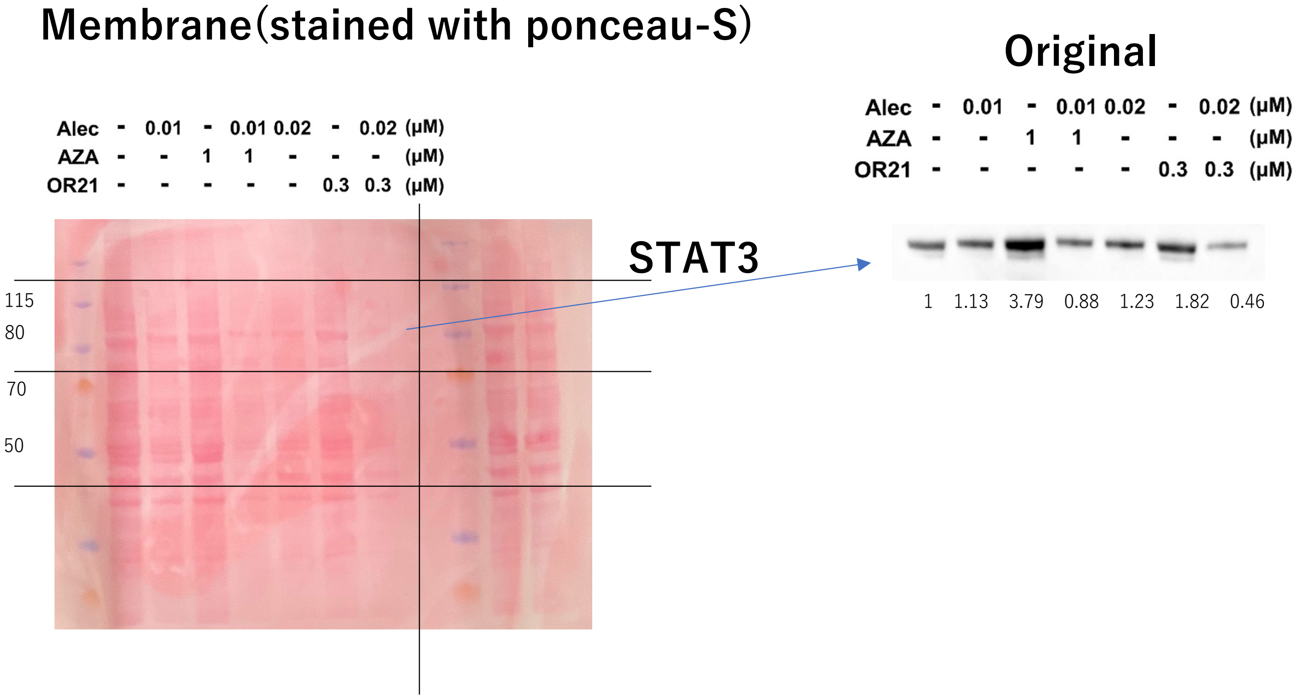

Membrane(stained with ponceau-S)
Alec - 0.01 - 0.01 0.02 - 0.02 (μM)
AZA - - 1 1 - - - (μM)
OR21 - - - - - 0.3 0.3 (μM)
115
80
70
50
STAT3
Original
Alec - 0.01 - 0.01 0.02 - 0.02 (μM)
AZA - - 1 1 - - - (μM)
OR21 - - - - - 0.3 0.3 (μM)
1 1.13 3.79 0.88 1.23 1.82 0.46
