# Supplementary material for: A Combination of Alectinib and DNA-Demethylating Agents Synergistically Inhibits Anaplastic-Lymphoma-Kinase-Positive Anaplastic Large-Cell Lymphoma Cell Proliferation
Source: Cancers (Basel). 2023 Oct 21;15(20):5089. doi: 10.3390/cancers15205089 (PMC10605931; doi:10.3390/cancers15205089)
Supplement: Supplementary file 1 [file cancers-15-05089-s001.zip › cancers-2402547-supplementary figures.pdf]

Supplementary information

A Combination of Alectinib and DNA-Demethylating Agents Synergistically Inhibits Anaplastic-Lymphoma-Kinase-Positive Anaplastic Large-Cell Lymphoma Cell Proliferation

Kazunori Kawasoe [1,2], Tatsuro Watanabe [1,*], Nao Yoshida-Sakai [1,2], Yuta Yamamoto [1,2], Yuki Kurahashi [1,3], Keisuke Kidoguchi [1,2], Hiroshi Ureshino [1,2], Kazuharu Kamachi [1,2], Yuki Fukuda-Kurahashi [1,3] and Shinya Kimura [1,2,*]

[1] Department of Drug Discovery and Biomedical Sciences, Faculty of Medicine, Saga University, Saga 849-8501, Japan
[2] Division of Hematology, Respiratory Medicine and Oncology, Department of Internal Medicine, Faculty of Medicine, Saga University, Saga 849-8501, Japan
[3] OHARA Pharmaceutical Co., Ltd., Koka 520-3403, Japan
* Correspondence: sn6538@cc.saga-u.ac.jp (T.W.); shkimu@cc.saga-u.ac.jp (S.K.);

Table of contents:

Supplementary methods
Supplementary Figures 1 to 6
Supplementary references

Supplementary methods

Quantitative real-time PCR

RNA extraction, synthesis of complementary DNA, and quantification of *SFRP5, CEL, GPR171,* and *ACTB* gene expression were performed using the TaqMan Gene Expression Master Mix (Applied Biosystems), as described previously[1]. Expression of *ACTB* was used as an internal control.

Cluster analysis

The global methylation dataset of ALCL patients was obtained from Gene Expression Omnibus (GSE66881)[2]. The data originated from: 1) tumor tissue samples from ALK+ and ALK^{-} ALCL patients (a tumor content > 90% was histopathologically verified); and 2) blood samples from five healthy individuals, from which CD3^{+} T cells were isolated to generate control data. The methylation data were generated using Illumina Infinium HumanMethylation450 BeadChip. The methylation level of a CpG site was represented by a b value, which ranged from 0 (completely unmethylated) to 1 (completely methylated). Unsupervised hierarchical clustering analysis was performed using the Heatplus package of R v3.4.4., as described previously[1,3].

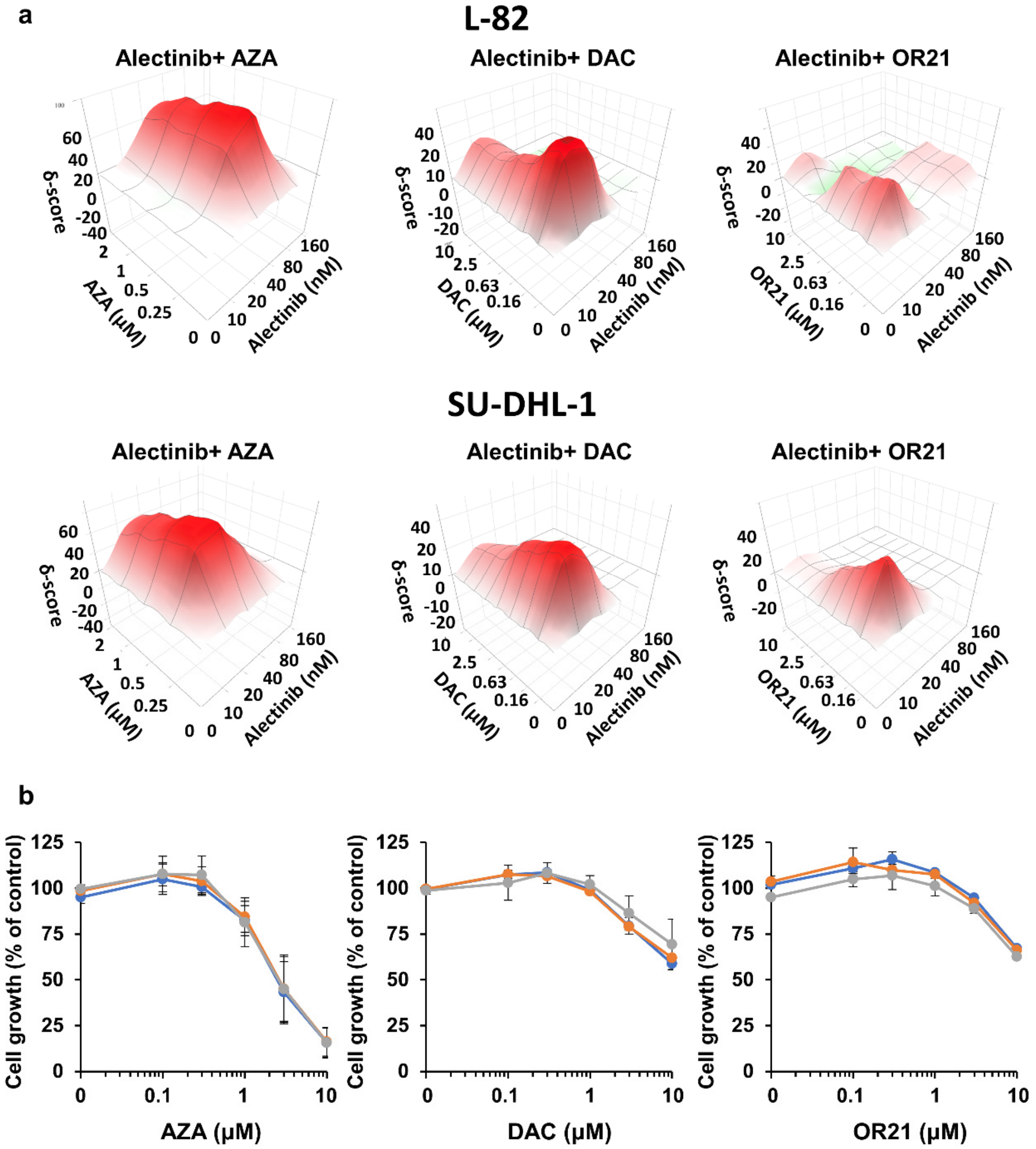

Supplementary Figure S1. Synergistic inhibition of cell proliferation induced by the combination of alectinib and DNA demethylating agents. a. ALK+ ALCL cell lines were treated with each compound alone or in combination for 4 days. Cell proliferation was determined using the CCK-8 assay and the synergy scores and maps were generated using the SynergyFinder web-application. **b.** Human primary hepatocytes were treated with different concentrations of DNA demethylating agents (AZA, DAC, or OR21) with/without 100 nM crizotinib or alectinib for 4 days (DNA demethylating agent alone: blue, combination with 100 nM crizotinib: orange, combination with 100 nM alectinib: grey). Cell proliferation was determined using the CCK-8 assay and the proliferation value of untreated cells was set at 100%. The results are expressed as the mean ± SD from three independent experiments.

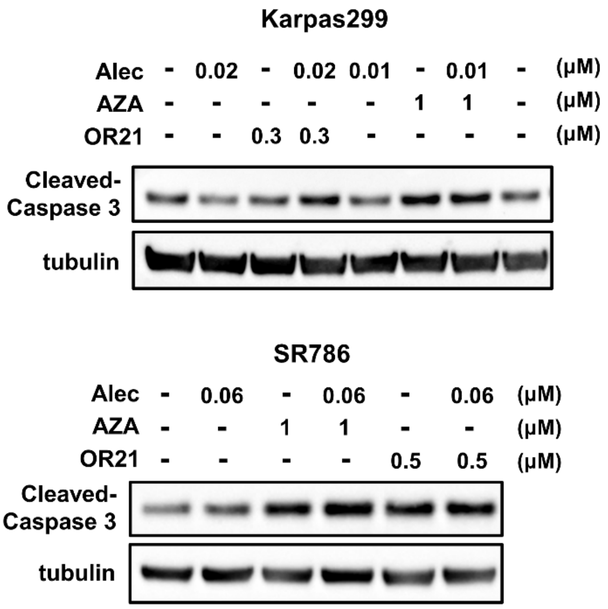

Supplementary Figure S2. Induction of cleaved caspase-3 protein by the combination of alectinib and DNA demethylating agents. Karpas299 and SR-786 cells were treated with each compound alone or in combination for 4 days. Immunoblots showing the amount of cleaved caspase 3 in cells exposed to the indicated treatment conditions.

a

	Ctr.	OR21	Alec	Alec OR21	AZA	Alec	Alec AZA
TNFRSF8	1.00	0.61	0.94	0.41	1.05	1.90	1.82
IL10	1.00	1.48	0.03	0.02	1.98	0.24	0.21
CD274	1.00	1.57	0.02	0.08	1.32	0.02	0.02
TGFB1	1.00	1.20	1.13	0.91	1.28	1.07	1.12
VEGFA	1.00	1.23	0.11	0.18	1.20	0.22	0.16
BCL2A1	1.00	0.67	0.02	0.04	0.93	0.01	0.02
MCL1	1.00	1.06	0.71	0.66	0.93	0.61	0.68
IL10RA	1.00	1.50	15.36	6.50	1.57	6.07	6.36
PDGFRA	1.00	0.88	0.00	0.04	1.15	0.38	0.23
PDGFRL	1.00	0.74	0.08	0.30	1.08	0.09	0.10
IRF4	1.00	0.58	3.50	0.66	0.86	3.40	2.72
CD276	1.00	1.77	0.52	0.30	1.48	0.56	0.45
HIF1A	1.00	1.78	0.58	0.46	1.49	0.59	0.48
MYC	1.00	0.66	0.11	0.13	0.95	0.87	0.56
BCL2L1	1.00	1.02	0.77	0.79	0.86	0.62	0.71
CCND1	1.00	25.25	0.00	0.50	9.25	0.25	0.00
NOTCH1	1.00	1.36	0.21	0.51	1.45	0.35	0.38

b

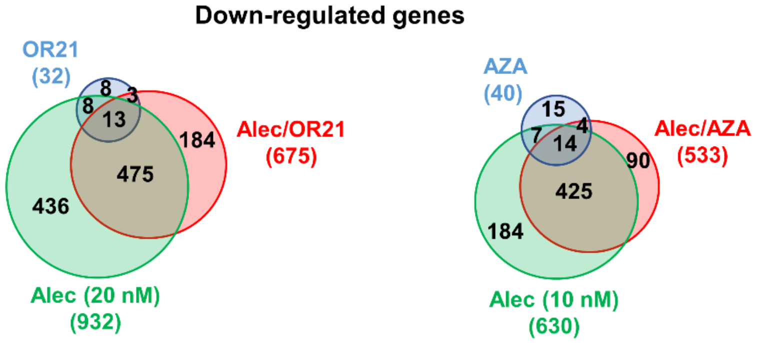

Supplementary Figure S3. Changes in gene expression profiles induced by the combination of alectinib and DNA demethylating agents. a. Karpas299 cells were treated with each compound alone or in combination for 3 days. Normalized expression levels (transcripts per million) of downstream target genes of ALK obtained from the analysis of RNA-seq data are shown as relative expression levels (compared to control in each gene). **b.** Venn diagrams show the number of down-regulated (≤ 0.2 fold change) genes relative to the control in the transcriptome analysis.

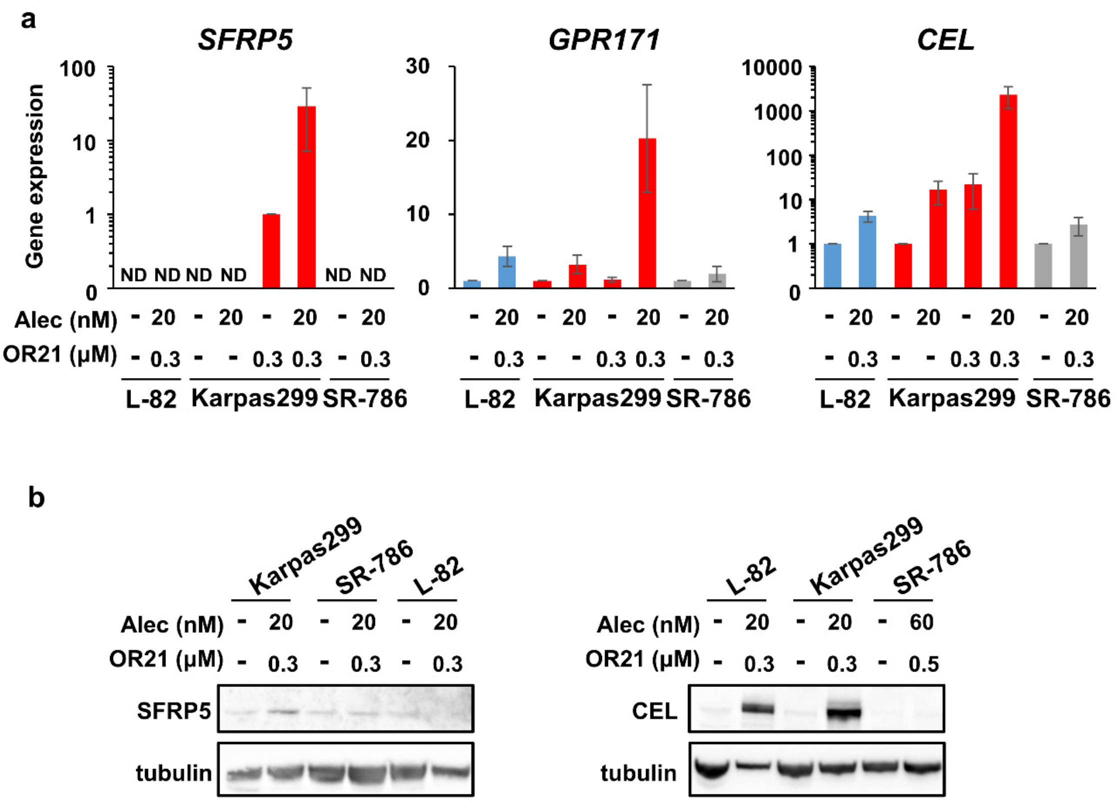

Supplementary Figure S4. Up-regulation of *SFRP5*, *GPR150,* and *CEL* gene expression induced by the combination of alectinib and OR21. ALCL cell lines were treated with each compound alone or in combination for 3 days. Gene expression levels of *SFRP5*, *GPR171*, and *CEL* was determined by real-time PCR. For the L-82 and SR-786 cell lines, relative gene expression is shown as a fold change relative to that of each of the untreated cells after normalization against *ACTB* gene expression levels. Since the *SFRP5* expression was not detected in untreated Karpas299 cells, relative gene expression is shown as a fold change relative to that in Karpas299 cells treated with OR21 alone. The data represent the averages of the results of more than three independent experiments. **b.** Immunoblots showing the amount of SFRP5 and CEL in cells exposed to the indicated treatment conditions.

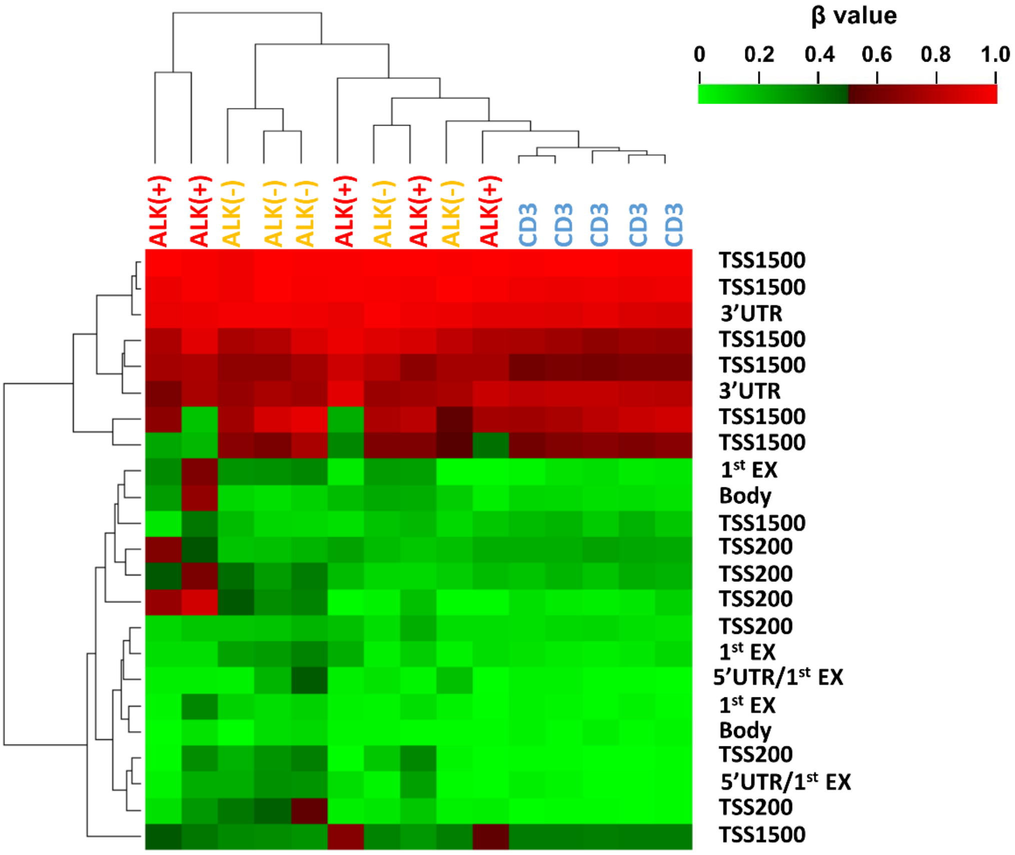

Supplementary Figure S5. Promoter hypermethylation of *SFRP5* in ALCL cells. Unsupervised hierarchical clustering analysis of the *SFRP5* gene methylation status in $CD3^+$ T cells isolated from the blood of healthy individuals (CD3) or in ALCL cells derived from the tumor tissues of patients with ALK+ ALCL (ALK+) and ALK− ALCL (ALK−). The methylation dataset was obtained from the Gene Expression Omnibus (NCBI GSE66881). TSS: transcription start site; 1st EX: first exon; Body: gene body.

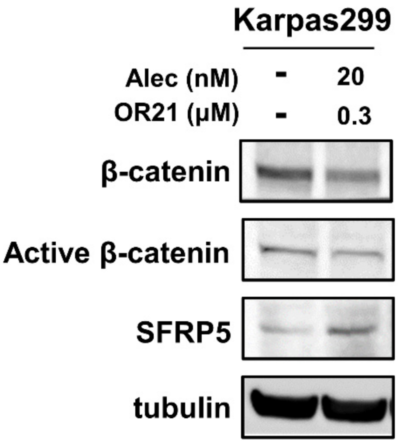

Supplementary Figure S6. Reduction of β-catenin associated with induction of SFRP5 by the combination of alectinib and OR21. Karpas299 cells were treated with OR21 and alectinib for 3 days. Immunoblots showing the amount of β-catenin, active β-catenin (non-phosphorylation at Ser45) and SFRP5 proteins.

Supplementary references

1. Watanabe, T., *et al.* Targeting aberrant DNA hypermethylation as a driver of ATL leukemogenesis by using the new oral demethylating agent OR-2100. *Blood* **136**, 871-884 (2020).
2. Hassler, M.R., *et al.* Insights into the Pathogenesis of Anaplastic Large-Cell Lymphoma through Genome-wide DNA Methylation Profiling. *Cell reports* **17**, 596-608 (2016).
